# Bibliometric analysis of *Helicobacter pylori* vaccine development from 1993 to 2023

**DOI:** 10.3389/fmicb.2025.1479195

**Published:** 2025-03-17

**Authors:** Yeqing Lei, Xiaochen Liu, Qin Du, Yan Li

**Affiliations:** ^1^Department of Gastroenterology, The Fourth Affiliated Hospital, Zhejiang University School of Medicine, Yiwu, China; ^2^Department of Gastroenterology, The Second Affiliated Hospital, Zhejiang University School of Medicine, Hangzhou, China

**Keywords:** *Helicobacter pylori*, vaccine, bibliometric analysis, visualization, treatment

## Abstract

**Background:**

*Helicobacter pylori* infects half the global population and imposes a huge health burden. Developing a vaccine targeting *H. pylori* appears to be the most ideal preventive option. Based on Web of Science Core Collection (WoSCC) publications from 1993 to 2023, this study visually analyses the current status and trends of this field through bibliometric analysis.

**Methods:**

*H. pylori* vaccine-related articles and reviews were retrieved from WoSCC. Microsoft Excel, CiteSpace, and VOS viewer were used to analyze the data.

**Results:**

1,199 publications from 1993 to 2023 were included in this bibliometric analysis. The results of this analysis show an overall upward trend in the number of publications and citations in this field. The United States is undoubtedly the most important contributor to this field in terms of publications, citation frequency, and national cooperation. *Vaccine* has the highest number of publications. Thomas F. Meyer is one of the leading scholars in the field. The most frequently cited article is “Immunization of mice with urease vaccine affords protection against *H. pylori* infection in the absence of antibodies and is mediated by MHC class II-restricted responses.” The literature and keyword analysis show that effective treatments and multi-epitope vaccines are focus area in this field. New antigen combinations (such as UreB, outer membrane vesicles, etc.) of *H. pylori* vaccines are novel research directions and frontiers.

**Conclusion:**

Our study is the first bibliometric analysis of *H. pylori* vaccine research. By summarizing the current status of *H. pylori* vaccine research, our study highlighted the current research direction and frontier, providing valuable data for researchers to grasp the latest advancements and accelerate *H. pylori* vaccine development.

## 1 Introduction

*Helicobacter pylori* (*H. pylori*) is a microaerophilic, gram-negative, flagellated, curved-rod bacterium that infects half the global population (Malfertheiner et al., 2022). *H. pylori* infection has been proven to be a crucial factor in the development of several gastrointestinal diseases, such as chronic gastritis, peptic ulcers, and mucosa-associated lymphoid tissue lymphoma (MALT), notably as the main risk factor for the development of gastric cancer (Robinson and Atherton, 2021). Moreover, there is substantial evidence linking *H. pylori* to numerous extra-gastric diseases, emphasizing the importance of implementing effective preventive and treatment strategies against this bacterium (Robinson and Atherton, 2021; Tsay and Hsu, 2018). As early as 1994, the World Health Organization (WHO) International Agency for Research on Cancer classified *H. pylori* as a Class I carcinogen for gastric cancer, acknowledging its significant role in the development of this deadly disease (Working Group on the Evaluation of Carcinogenic Risks to Humans, 1994). *H. pylori* can be transmitted through multiple pathways, including fecal-oral, oral-oral, and gastric-oral, allowing for both human-to-human and animal-to-human transmission (Duan et al., 2023). A recent meta-analysis revealed significant regional variations in *H. pylori* infection rates, ranging from 18.9% in Switzerland to 87.7% in Nigeria, attributable to geographical, economic, and social factors (Hooi et al., 2017). Studies have also identified several independent risk factors for childhood *H. pylori* transmission, including residing in high-infection areas, belonging to large families, and having infected family members, particularly mothers (Zhou et al., 2023; Ding et al., 2022). Therefore, according to the current Maastricht VI/Florence consensus report, all *H. pylori*-positive patients should receive treatment regardless of clinical symptom (Malfertheiner et al., 2022). Since it was first observed by Barry Marshall and Robin Warren in 1983, a combination of antibiotics and antacids has been developed for its eradication (Malfertheiner et al., 2022; Warren and Marshall, 1983). Empirical treatment with bismuth quadruple therapy and the current hot high-dose dual therapy has achieved promising eradication rates in the adult population; however, the increasing prevalence of antibiotic resistance presents a significant obstacle in the ongoing efforts to eradicate *H. pylori* (Malfertheiner et al., 2022; Qian et al., 2023; Tshibangu-Kabamba and Yamaoka, 2021). In the past decade, there has been a notable increase in global resistance levels of *H. pylori* to key antibiotics like clarithromycin, metronidazole, and levofloxacin, reaching concerning rates of ≥15% worldwide (Savoldi et al., 2018).

*H. pylori* has evolved numerous mechanisms to colonize the hostile acidic environment of the human stomach successfully; these mechanisms include flagellar motility for movement, chemotaxis signaling for environmental sensing, urease synthesis, and ammonia production for pH neutralization, bacterial toxins for host cell manipulation, and adhesion molecules for attachment to the gastric mucosa (Toh and Wilson, 2020). Several molecules with potential as vaccine candidates, such as urease, CagA, VacA, and BabA, play crucial roles in these mechanisms (Dos Santos Viana et al., 2021). The development of vaccines targeting *H. pylori* offers a promising alternative for achieving global eradication of this pathogen. However, current research in this area remains highly challenging. Most research is still in its early stages and encounters significant obstacles, particularly the need to unravel the immunotolerance mechanism of *H. pylori* (Li et al., 2023).

Currently, the research direction and focus areas in this field remain ambiguous. Therefore, we are conducting a literature analysis on *H. pylori* vaccine research, aiming to fully leverage the existing research foundation and pinpoint the essential areas of concentration within this field. Bibliometric analysis is a novel scientific approach that utilizes a blend of mathematical and statistical techniques to assess the productivity of countries, institutions, authors, and journals within a specific research field (Shi et al., 2022). These information can be used by researchers to visualize the basics and focus areas of the research field, as well as to forecast emerging trends and research frontiers (Chen et al., 2012).

Previous bibliometric analyses of research focus areas, geographical distribution, and temporal trends on *H. pylori* and drug resistance, microbiota, and immunotherapy have been published (Shi et al., 2022; Li et al., 2023; Li et al., 2023). However, there is still a lack of comprehensive and detailed literature data statistics and analysis on *H. pylori* vaccine-related research. On this ground, a comprehensive analysis encompassing both qualitative and quantitative aspects of articles on *H. pylori* vaccines is imperative. The bibliometric research on this topic will not only enhance the existing knowledge in the field, offer crucial insights into the discipline, but also uncover intricate interconnections among various research directions within the field. This bibliometric analysis, covering a total of 1,199 studies conducted from 1993 to 2023, presents the current status and future trends in *H. pylori* vaccine research, offering more detailed data and introducing new perspectives to stimulate further research endeavors into the development of effective *H. pylori* vaccines.

## 2 Methods

### 2.1 Data collection

In this study, the data were obtained from the Web of Science Core Collection (WoSCC). WoSCC is an online database that provides standardized and up-to-date reference data sets for use in scientific research. The search strategy combined the topics of *H. pylori* and vaccine using the following search formula: (TS = (“*Helicobacter pylori*” OR “*Campylobacter pylori*” OR “*H. pylori*” OR “*Campylobacter pylori subsp. Pylori*” OR “*Campylobacter pyloridis*” OR “*Campylobacter pylori*”)) AND TS = (Vaccine). As of June 3, 2024, a total of 1,199 original English-language articles on *H. pylori* and vaccines are retrieved from 1993 to 2023, encompassing both articles and reviews. The study selection process is shown in a flow diagram (Supplementary Figure S1).

### 2.2 Data analysis

The complete records of retrieved papers, including title, author, country, journal, keywords, institutions, and references, were exported as a plain text file and then were imported into Microsoft Excel 2016 (Microsoft, Washington, United States), CiteSpace (version.6.3. R1 Advanced), and VOS viewer (version.1.6.20) for qualitative and quantitative analyses. Microsoft Excel 2016 has been used for database management and the analysis of annual publications. CiteSpace, a Java-based information visualization program created by Professor Chen (Drexel University), was used to analyze the reference collaboration, dual maps of journals, reference bursts, and keyword bursts (Chen and Song, 2019). VOS viewer was developed by Nees Jan van Eck et al. and is used for co-citation and co-occurrence analysis, and to construct and visualize the literature network map (van Eck and Waltman, 2010).

## 3 Results

### 3.1 Publication numbers and citation trends

**Figure 1:**
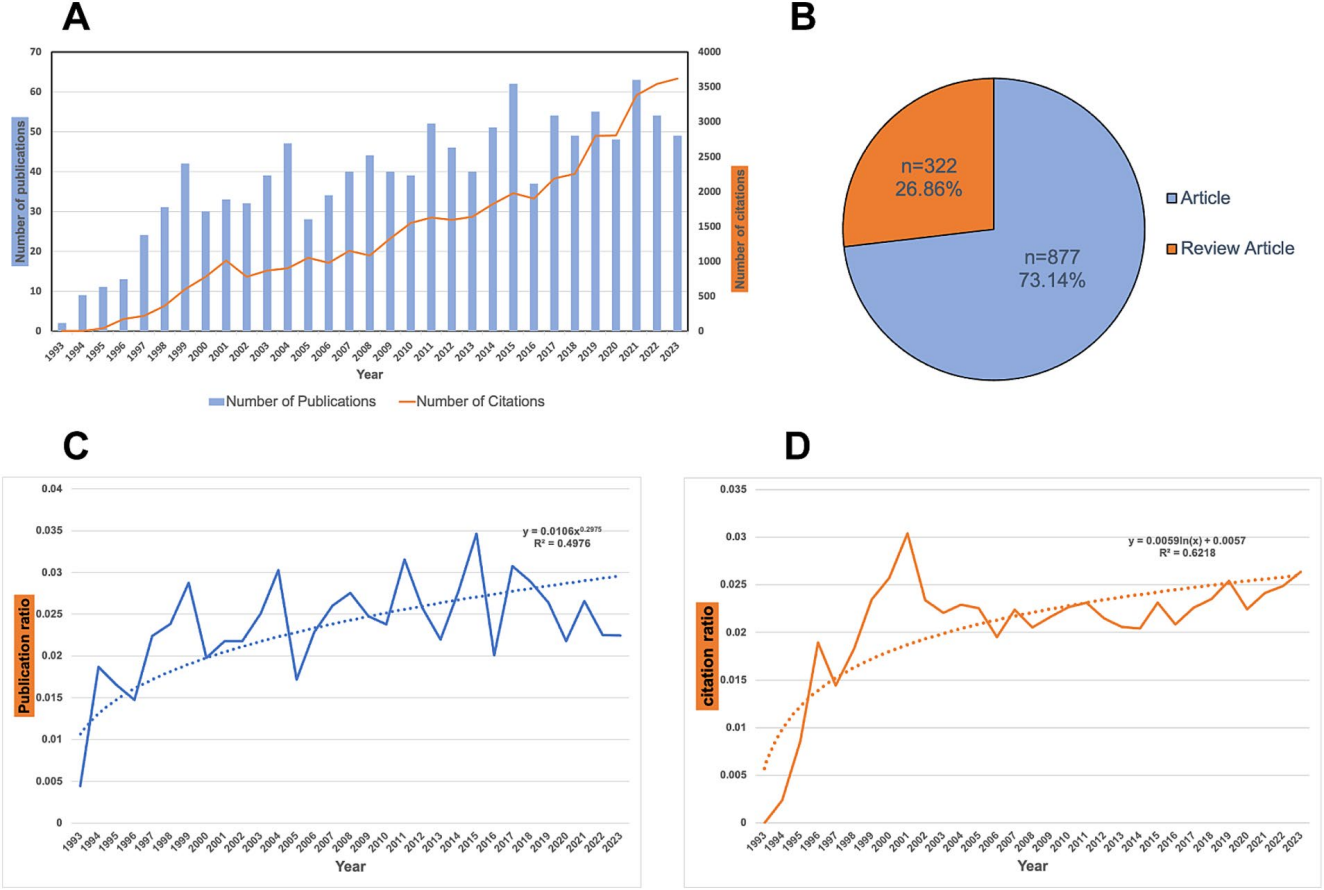
Analysis of publications related to *H. pylori* vaccine. **(A)** Trend in the publications related to *H. pylori* vaccine research from 1993 to 2023. **(B)** Document types composition summary. A total of 1,199 studies were analyzed. Of those, 877 were original research articles, and 322 (26%) were reviews. **(C)** Trend in the publication ratio related to *H. pylori* vaccine research from 1993 to 2023. We employ the following search formula: (TS = (“*Helicobacter pylori*” OR “*Campylobacter pylori*” OR “*H. pylori*” OR “*Campylobacter pylori subsp. Pylori*” OR “*Campylobacter pyloridis*” OR “*Campylobacter pylori*”)) to search the English- language literature on *H. pylori* from 1993 to 2023 in the WoSCC database, encompassing articles and reviews. We record the number of publications and citations each year. Subsequently, we calculate the ratio between *H. pylori* vaccine publications and general *H. pylori* research. The curve depicts the change in the ratio of the number of *H. pylori* vaccine-related studies to general *H. pylori* studies from 1993 to 2023. The dashed line indicates the tendency in the proportion of the number of studies related to the *H. pylori* vaccine to the number of general *H. pylori* studies. **(D)** Trend in the citation ratio related to *H. pylori* vaccine research from 1993 to 2023. The curve shows the change in the ratio of citations from *H. pylori* vaccine-related studies to those from general *H. pylori* studies between 1993 and 2023. The dashed line shows the tendency in the proportion of *H. pylori* vaccine-related citations in general *H. pylori* research.

1,199 papers were retrieved from the WoSCC database. Figure 1A shows the annual trends in publication output and citation frequency for articles related to *H. pylori* vaccine research. The publication number in each period serves as an indicator of the research advancements and trends in the field of *H. pylori* vaccine development. As shown in Figure 1A, the number of publications on *H. pylori* vaccine remained low from 1993 to 2023, with an overall increasing trend. Based on the peak curve of publications, three main stages can be identified. (1) 1993–1999: representing the initial phase of *H. pylori* vaccine development, accounting for 11.0% (*n* = 132) of the total publications and peaking in 1999 with 42 articles; (2) 2000–2016: characterized by fluctuating publication outputs related to *H. pylori* vaccine development, accounting for 57.9% (*n* = 694) of total publications; and (3) 2017–2023: indicating a stable phase in *H. pylori* vaccine development with an average annual publication output ranging from 48 to 63 articles per year. Notably,2021 has the highest number of publications (63), while the highest number of citations occurred in 2023 (3617), with an average of 38.6 papers published per year and 36.7 citations per paper. Of these, 877 (73.14%) were original research articles, and 322 (26.86%) were review articles (Figure 1B).

Additionally, we employ the following search formula: (TS = (“*Helicobacter pylori*” OR “*Campylobacter pylori*” OR “*H. pylori*” OR “*Campylobacter pylori subsp. Pylori*” OR “*Campylobacter pyloridis*” OR “*Campylobacter pylori*”)) to search for the English-language literature related to *H. pylori* from 1993 to 2023 in the WoSCC database, including articles and reviews. Subsequently, we record the number of publications and citations each year of the general *H. pylori* research and, respectively, calculate the ratios between *H. pylori* vaccine publications/citations and the general *H. pylori* research. These data are presented in Figures 1C,D. As can be observed from Figure 1C, the trend line is generally increasing (*R*^2^ = 0.4976), yet the ratio between the number of *H. pylori* vaccine-related studies and the number of general *H. pylori* studies is unstable. The trend in terms of the number of publications on *H. pylori* vaccine research has not exceeded that of general *H. pylori* research. Nevertheless, we can still notice an overall increase in the number of *H. pylori* vaccine studies from 1993 to 2023. In Figure 1D, the proportion of citations for *H. pylori* vaccine studies is increasing from year to year, both from the ratio curve and the trend line (*R*^2^ = 0.6218). We can infer that the development trend of the number of citations in *H. pylori* vaccine-related studies surpasses that in general *H. pylori* studies.

### 3.2 Countries and institutions contribution

The 1,199 papers were contributed by researchers from 82 different countries. To highlight the most influential nations, Supplementary Table S1 illustrates the top 10 countries based on their contributions in the number of publications, citations, and total link strength. Link strength is utilized to denote the frequency of cooperation among countries. A higher value implies more cooperation with other countries. Notably, China, the United States, and the United Kingdom are ranked in the top three positions for both publications and total link strength. Meanwhile, Italy, the United States, and the United Kingdom are ranked in the top three for citation frequency. In terms of number of publications, only the United States (327) has over 300, followed by China (236) and the United Kingdom (92). In terms of citations, the United States still holds the top position with 18,360 citations, followed by Italy in second place with 6,071 citations, and the United Kingdom in third place with 4,720 citations. In terms of total link strength ranking, the United States is ranked first with a score of 194, followed by the United Kingdom with a score of 90, and China with a score of 67.

**Figure 2:**
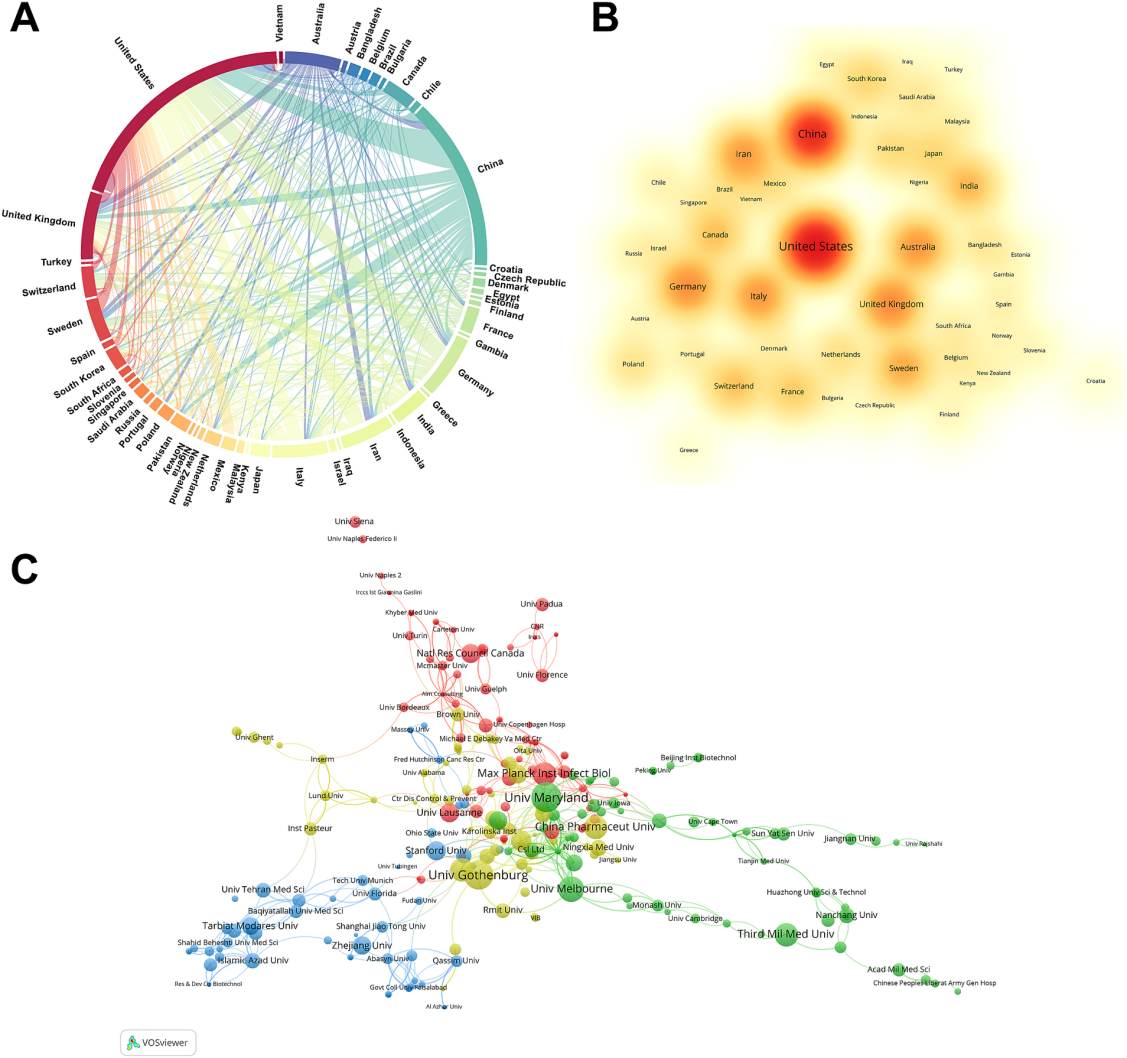
Countries and institutions analysis. **(A)** Country co-occurrence map of *H. pylori* vaccine. The arc length directly correlates with the volume of publications from each country, and the thickness of the connection reflects the strength of the partnership. **(B)** The density visualization map of countries in *H. pylori* vaccine research. The magnitude of the word, the magnitude of the round, and the opacity of red are positively correlated with the number of publications. **(C)** *H. pylori* vaccine relevant institutions cluster analysis. Each node represents an institution, and the size of the circle is proportional to the number of articles published by that institution. The connections between nodes indicate the degree of correlation, and a larger number of connections implies more cooperation.

In Figure 2A, a chord diagram visually represents the collaborative relationship among countries. The arc length directly correlates with the volume of publications from each country, and the thickness of the interconnecting chords indicates the strength of their collaborative relationship. The United States ranks first in terms of the number of publications, followed by China and the United Kingdom. Meanwhile, the United States has the closest relationship with China, while also engaging in the most extensive collaboration with other countries. Figure 2B offers a density visualization map of nations, which could highlight high-publication countries clearly. Overall, the United States and China had the hottest color for the number of publications.

A total of 1,371 institutions from various countries participated in 1199 studies. Supplementary Table S2 shows the top 10 institutions based on their publications and citation frequency. Four are from the United States, two from China, and one each from Sweden, Australia, Germany, and Switzerland. The University of Maryland, with 36 publications, has the highest publications, closely followed by the University of Gothenburg (34 publications) and the University of Melbourne (26 publications). Universities in the United States and China have become pivotal players in the field of *H. pylori* vaccine research, contributing significantly to advancements in the field. In the ranking of citation frequency, The Stanford University ranked first with 1968 citations, followed by the University of Maryland with 1716 citations and the Max Planck Institute for Infection Biology with 1,529 citations. Meanwhile, we conducted a cluster analysis of these institutions to gain insight into the collaboration between institutions on *H. pylori* vaccine research. Figure 2C shows a network map of institutions with a frequency of ≥2 publications, divided into four clusters by color. The red cluster is the largest network of collaborating institutions, which includes the Max Planck Institute for Infection Biology, the University of Lausanne, and the National Research Council of Canada. The second cluster (green cluster) is represented by the University of Maryland, the University of Melbourne, and the Third Military Medical University. The third cluster (blue cluster) mainly comprises the Stanford University, Zhejiang University, and Tarbiat Modares University. The fourth cluster (yellow cluster) mainly comprises the University of Gothenburg, China Pharmaceutical University, and Harvard University.

### 3.3 Author and co-cited author analysis

**Figure 3:**
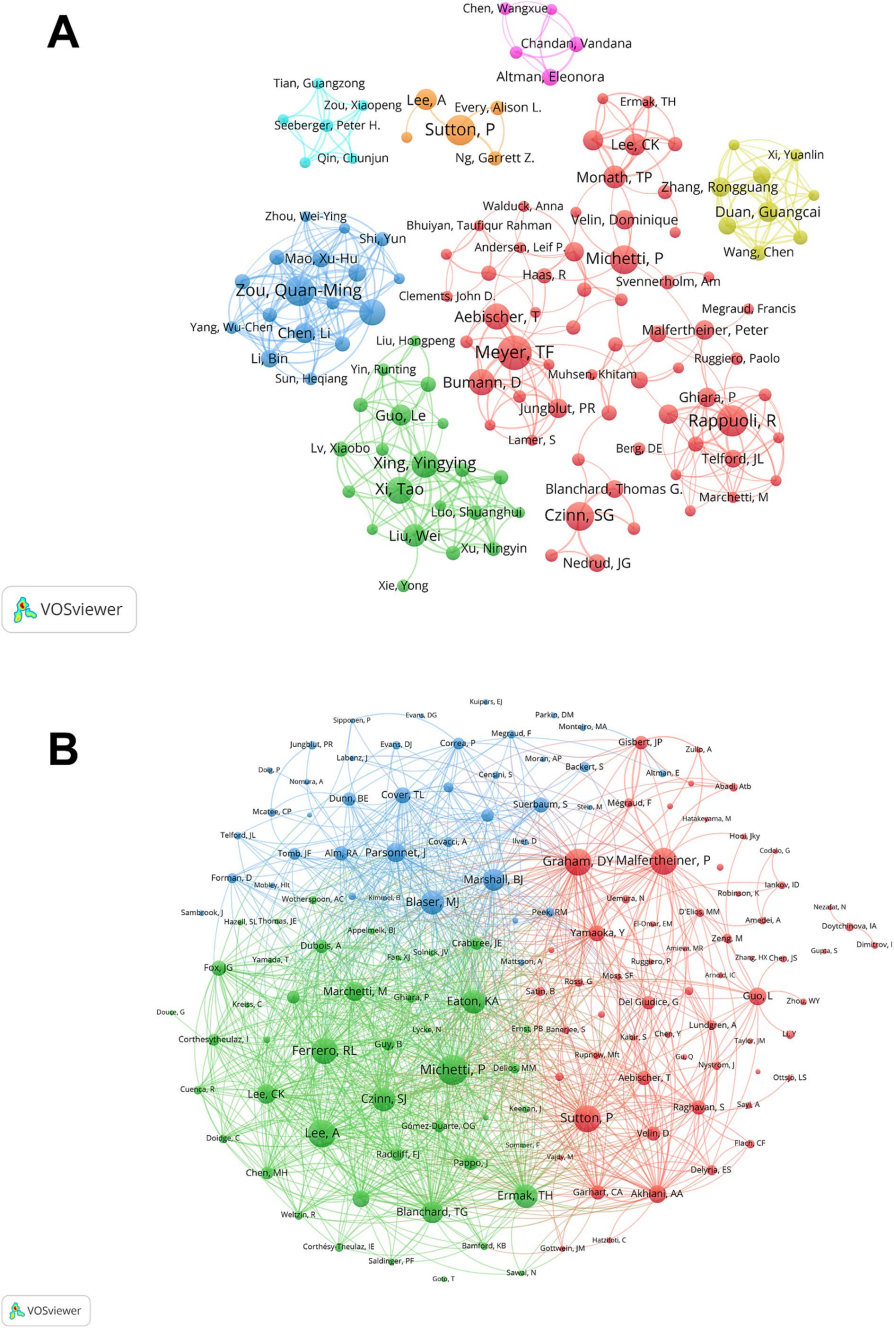
The author and co-cited author contribution analysis. **(A)** The co-author map in the field of *H. pylori* vaccine. Each circle represents a distinct author, and the connecting lines between the circles indicate the interrelationships among the authors. The nodes of the same color denote the same cluster. **(B)** The co-cited authors map in the field of *H. pylori* vaccine. Nodes of the same color pertain to the same cluster. Nodes of distinct colors denote the authors of various collaborative relationships. The magnitude of the word, the size of the circle, and the thickness of the connection are positively correlated with the co-citation frequency.

Over 4,000 authors have contributed to research on *H. pylori* vaccines, and by analyzing the authors, we can distinguish key contributors and leading countries in this field. Supplementary Table S3 shows the top 10 productive authors in this field along with their respective countries. Among the top 10 authors, Thomas F. Meyer takes the lead with 28 publications, closely followed by R. Rappuoli (23), Philip Sutton (22), and Quan-Ming Zou (22). The remaining six authors each boast over 15 publications, demonstrating their substantial contributions to this field. Notably, three of the ten are from Germany and another three are from China. Figure 3A illustrates the co-authors cluster analysis. Collaboration among scholars in this field appears to be relatively dispersed, lacking close and widespread connections. Thomas F. Meyer, serving as an active node in the red network, exhibits the broadest scope and occupies a relatively central position, indicating his extensive and dynamic collaborations with various authors in the field.

We then performed a statistical and cluster analysis of co-cited authors related to *H. pylori* vaccines, as depicted in Supplementary Table S3 and Figure 3B. Among the top 10 authors, only Pierre Michetti had a co-citation count exceeding 300 and a total link strength surpassing 7,000. Additionally, seven authors had total link strengths exceeding 6,000, and two had strengths over 5,000. An interesting observation is that five of the top 10 co-cited authors are from the United States, underscoring the country’s significant contribution to the field. Pierre Michetti leads the pack with 309 co-citations, closely followed by A Lee (271) and Peter Malfertheiner (269). Additionally, Pierre Michetti has a total link strength of 7,855, ranking first, followed by Richard L. Ferrero (6875), and Thomas G. Blanchard (6834). It is noteworthy that both Pierre Michetti and Philip Sutton are ranked in the top 5 for both the publications and co-citations, highlighting their significant contributions and widespread recognition in this field. Figure 3B shows a cluster analysis of co-cited authors, visually illustrating the network of academic partnerships and collaborations in this field. Nine out of the top 10 co-cited authors occupy the most active positions in the red and green clusters, which indicates that the research direction of the authors with high co-citations is mainly divided into two directions, but the connection between them remains close.

### 3.4 Journal and co-cited journal analysis

**Figure 4:**
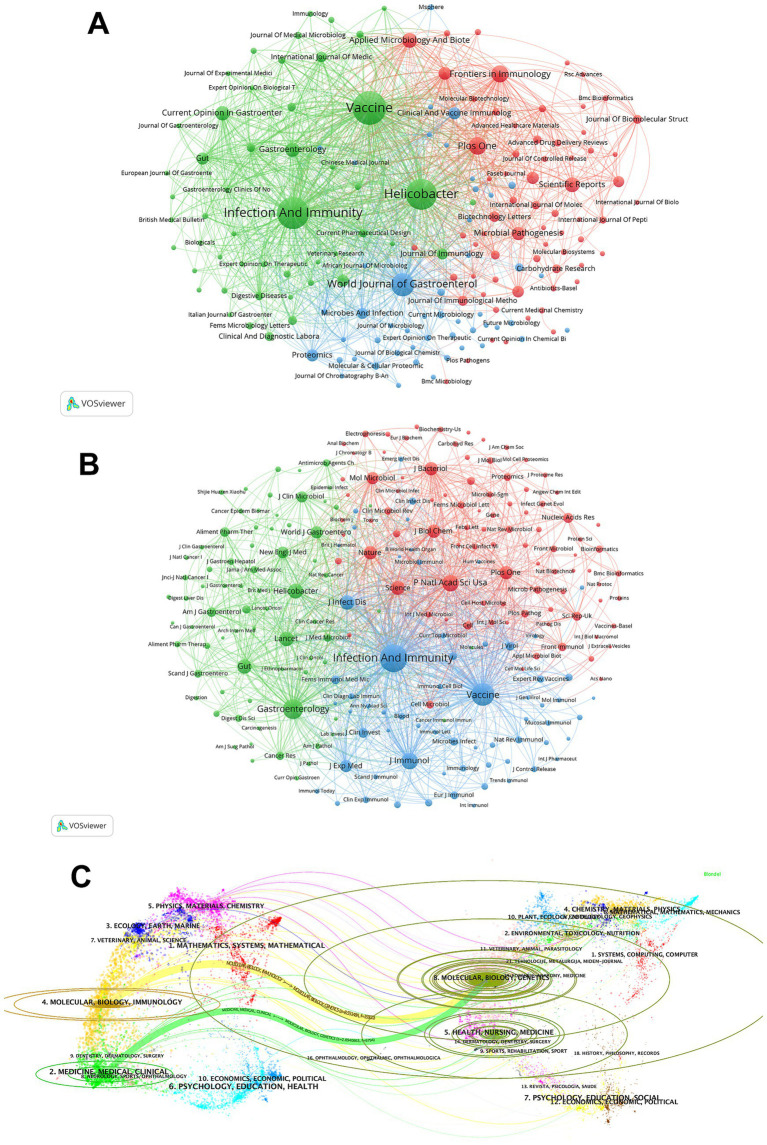
The journal and co-cited journal contribution analysis. **(A)** The journal map in the field of *H. pylori* vaccine. Each node symbolizes a journal. Nodes of the same color indicate that they pertain to the same cluster. The size of the node represents the number of publications, and the link denotes the citation relationship between journals. The thickness of the link reflects the citation intensity between journals. **(B)** The co-cited-journal map in the field of *H. pylori* vaccine. Nodes of the same color belong to the same cluster. The size of the node indicates the co-citation frequency of the journal, and the link represents the co-citation relationship between journals. The thickness of the link reflects the co-citation strength between journals. **(C)** The dual-map overlay of journals on *H. pylori* vaccine. The labels situated on the left side of the dual map stand for citing journals, while those on the right represent cited journals. The colored paths denote citation relationships.

A total of 462 journals from different countries published articles on *H. pylori* vaccine. The top 10 most productive journals in terms of publications and co-citations are shown in Supplementary Table S4. All of the top ten journals are indexed in either Q1 or Q2 JCR, with five of them having an IF exceeding 4. *Vaccine* and *Infection And Immunity* have each published over 70 articles, indicating their prominence in this field compared to other journals. In terms of publication volume, the top five most productive journals are *Vaccine* (82), *Infection And Immunity* (74), *Helicobacter* (67), *World Journal of Gastroenterology* (39), and *Plos One* (23). These top five journals collectively accounted for 285 publications, representing 23.77% of the total. We then employ cluster analysis to categorize journals into three groups, as depicted in Figure 4A. Nodes with the same color indicate that they belong to the same cluster. The size of the node represents the number of publications, and the link denotes the citation relationship between journals. The thickness of the link reflects the citation intensity between journals. We observe that there are numerous close and extensive connections within the journal network. The top three journals in terms of article number all belong to the green zone. The red group contains journals such as *Plos One* and *Frontiers in Immunology*. The blue cluster covers journals such as *World Journal of Gastroenterology* and *Proteomics*.

Supplementary Table S4 highlights the top 10 co-cited journals, all of which are positioned within Q1 JCR or Q2 JCR. Four out of these ten journals exhibited an IF exceeding 20. *Infection And Immunity* holds the highest rank, with IF = 2.9 and 6,001 citations, followed by *Vaccine* (IF = 4.5, with 3,698 citations) and *Gastroenterology* (IF = 25.7, with 2,689 citations). *Infection And Immunity* and *Vaccine* are the only two journals that rank in the top 5 within both publications and citations. As shown in Figure 4B, we can directly observe the co-citation relationships between co-cited journals through cluster analysis and visualization. The top three positions in the ranking of co-cited journals are dominated by the most influential journals from the red, blue, and green clusters, suggesting a robust and interconnected citation network between these journals.

The dual-map overlay of journals presents the distribution of topics and the changes in citation trajectories within scientific journals (Figure 4C). The labels on the left side of the dual map represent citing journals and those on the right represent cited journals. The colored paths indicate citation relationships. Figure 4C shows the two main citation paths of the *H. pylori* vaccine. In this area, both the MOLECULAR/BIOLOGY/IMMUNOLOGY (orange line) and the MEDICINE/MEDICAL/CLINICAL (green line) journals, called research frontiers, are considerably influenced by the MOLECULAR/BIOLOGY/GENETICS journals, called the knowledge base. The strongest citation links come from the MOLECULAR/BIOLOGY/IMMUNOLOGY to the MOLECULAR/BIOLOGY/GENETICS journals.

### 3.5 Keyword analysis

**Figure 5:**
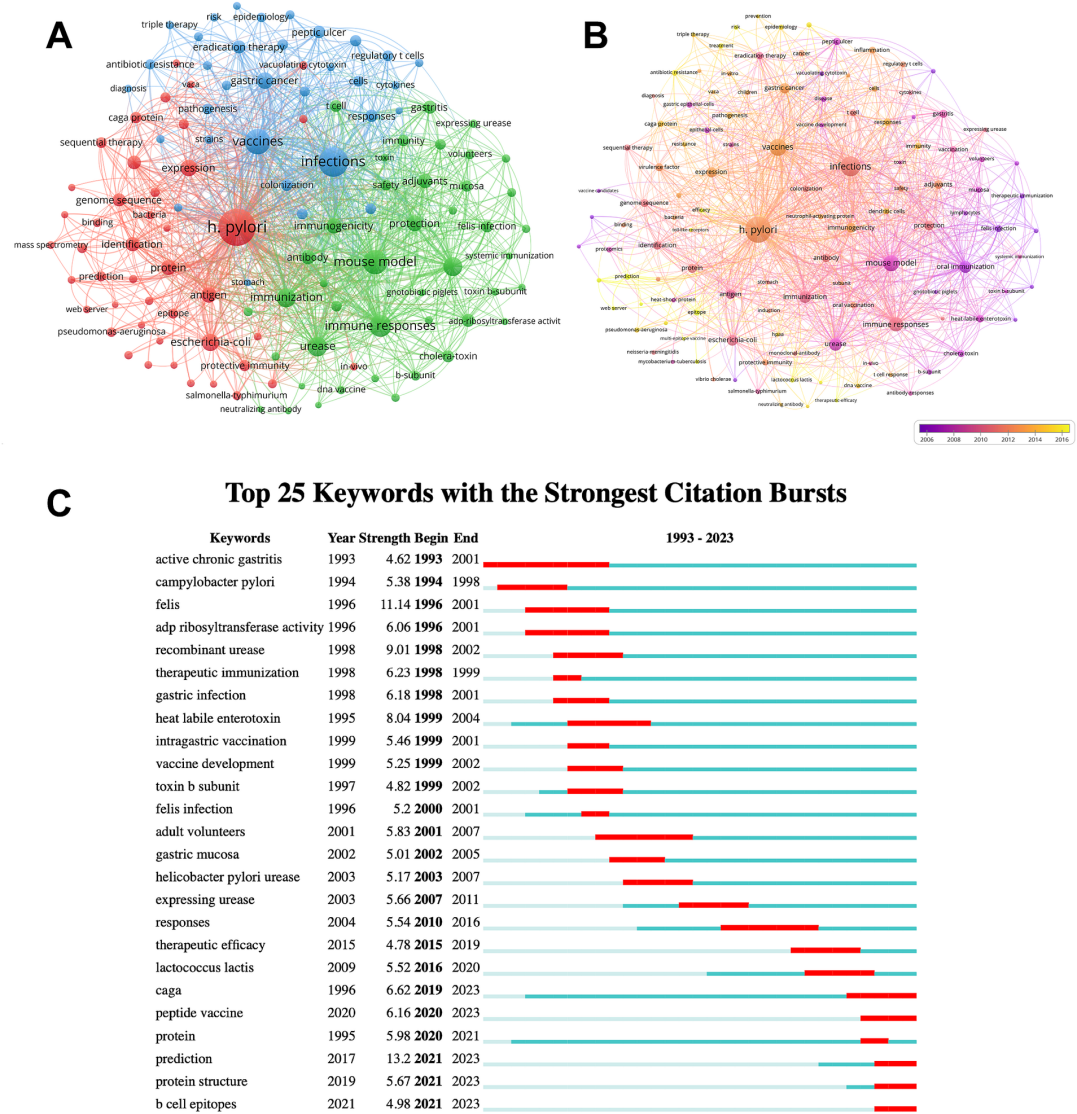
The keyword analysis. **(A)** The network visualization map of keyword co-occurrence analysis. Keywords are classified into three categories. Each keyword is presented as a node. The nodes of distinct colors represent different clusters and the size of nodes indicates their frequency. The lines connecting the nodes represent the co-occurrence relationship. **(B)** The overlay visualization map of keyword co-occurrence analysis. Different colors represent different mean published years. **(C)** Top 25 keywords with the strongest citation bursts. These keywords are sorted by starting year. The blue bars signify that the reference has been published; the red bars indicate citation burst.

The keywords provide a comprehensive overview of the article’s core content. The top 20 keywords associated with *H. pylori* vaccine research are presented in Supplementary Table S5. Three keywords had over 300 occurrences, while ten had more than 100. Four of them possessed a total link strength exceeding 2000, while the remaining had more than 400. *H. pylori* (743) and vaccines (318), as the keywords of the search formula, were ranked first and third, respectively, in terms of keyword occurrences. We then used the VOS viewer to conduct a cluster analysis of keywords and create network and overlay visualization maps. As can be seen in Figure 5A, the whole cluster analysis network consists of three closely related clusters with a strong co-occurrence relationship. The red cluster contains virulence factors and proteins associated with *H. pylori*, which can serve as potential antigens to develop an effective *H. pylori* vaccine, such as caga protein, vaca, and heat-shock protein. The green cluster revolves around key aspects of vaccine development, including key concepts such as mouse models, immune response, and immunogenicity, revealing the complex process of vaccine manufacturing. Finally, the blue cluster explores the correlation between this bacterium and other medical conditions, such as gastric cancer, peptic ulcers, and various treatments including triple therapy. Figure 5B displays the popular keywords of each year in different colors through keyword cluster analysis. Purple represents an earlier year of publication, while yellow signifies a more recent year of publication. Since 2016, the keywords “antibiotic resistance,” “therapeutic efficacy,” and “multi-epitope vaccine” have garnered the greatest attention, suggesting that effective treatments might be the latest focus area for *H. pylori*.

We further used CiteSpace to analyze the burst detection of keywords. Figure 5C lists the top 25 keywords with the strongest citation bursts. Among these keywords, the earliest is active chronic gastritis, closely followed by *campylobacter pylori*. Recent years have seen a surge in interest surrounding peptide vaccines and B cell epitopes. These keywords reflect the current focus areas and frontiers in *H. pylori* vaccine development. Consequently, we infer that multi-epitope vaccines are a future focus area for *H. pylori* research. Based on our findings, we deduce that multi-epitope vaccines will become a crucial research focus in the future of *H. pylori* studies.

### 3.6 Reference analysis

Supplementary Table S6 presents the top 10 most frequently cited references that specifically concentrate on the *H. pylori* vaccine. Among them, only one article received over 300 citations, five articles garnered more than 200 citations, and the remaining four articles obtained more than 100 citations. Additionally, a significant majority of articles (8 out of the 10) were published in or before 2001.

The paper titled “Immunization of mice with urease vaccine affords protection against *Helicobacter pylori* infection in the absence of antibodies and is mediated by MHC class II-restricted responses” published in *The Journal of Experimental Medicine* by Ermak et al. (1998) is the most frequently cited paper (344 citations). This article reveals that immunizing mice with urease in the absence of B cells and antibodies can achieve protection against *H. pylori*, which is mediated by MHC class II CD4 T cell. The latest published article is “Efficacy, safety, and immunogenicity of an oral recombinant *Helicobacter pylori* vaccine in children in China: a randomised, double-blind, placebo-controlled, phase 3 trial” by Zeng, Ming et al., which was published in *The Lancet* in 2015, with a total of 169 citations (Zeng et al., 2015). In this study, Zeng, Ming et al. accomplished Phase III clinical trials of a recombinant oral vaccine against *H. pylori* containing UreB and LTB components and demonstrated excellent protection in children. Additionally, we mapped out the annual citations of the top 10 highly cited references since their publication, as depicted in Supplementary Figure S2. The size and color of the circles represent the citation counts of these works. Specifically, larger circles and colors ranging from blue to red indicate higher citation rates and greater influence within the field. Notably, the study by Zeng Ming et al., published in *The Lancet* in 2015, has received significant citations since its release (Zeng et al., 2015). Furthermore, it is crucial to emphasize that the study by Naz, Anam et al., published in *Infection, Genetics and Evolution* in 2015, also received considerable attention (Naz et al., 2015). Conversely, citations for the other eight papers published in 2001 or earlier have witnessed a decline in recent years.

**Figure 6:**
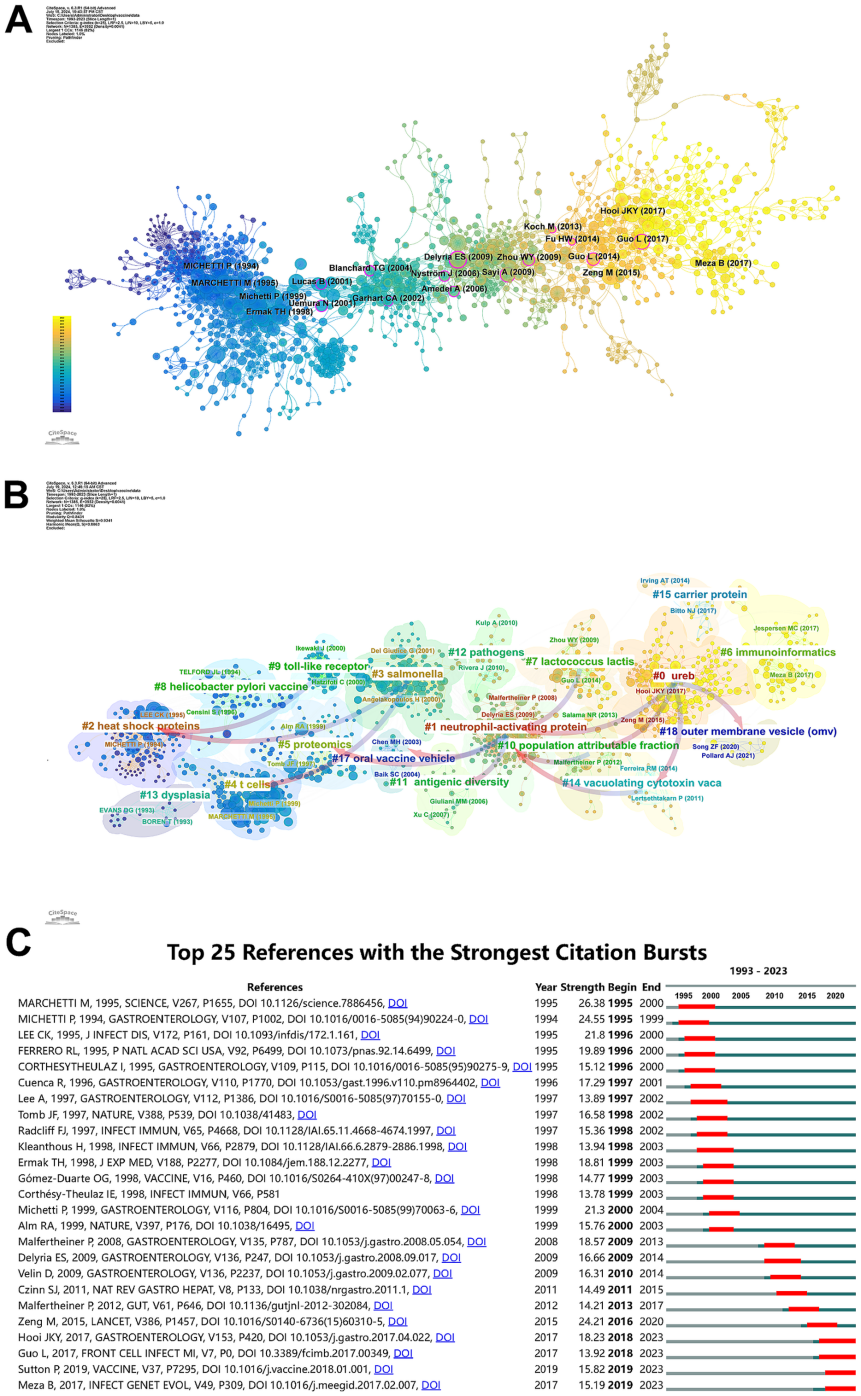
Analysis of *H. pylori* vaccine related references. **(A)** Co-cited references network related to *H. pylori* vaccine. The uniform color indicates that these references pertain to the same clusters, and the links denote the co-occurrence relationship among the references. The size of the node is in proportion to the frequency at which the article is co-cited. **(B)** Cluster view of references in *H. pylori* vaccine. Clustering of references based on similarity between references, including #0 UreB, #1 Neutrophil-activating protein, #2 Heat shock proteins, #3 *Salmonella*, #4 T cells, #5 Proteomics, #6 Immunoinformatics, #7 *Lactococcus lactis*, #8*Helicobacter pylori* vaccine, #9 Toll-like receptors, #10 Population attributable fraction, #11 Antigenic diversity, #12 Pathogens, #13 Dysplasia, #14 Vacuolating cytotoxin vaca, #15 Carrier protein, #17 Oral vaccine vehicle and #18 Outer membrane vesicle (OMV). **(C)** Top 25 references with the strongest citation bursts. The green bars mean the reference had been published; the red bars mean citation burst.

Figure 6A shows the main frequently co-cited references. Figure 6B shows the clustering relationship of references. These clusters are based on the degree of association between the literature and are distinguished by different colors. The most prominent research cluster (#0) has the largest publications, and the most common keyword in these articles is *H. pylori* urease B subunit (UreB), among which the primary citations were published by Zeng et al. (2015) and Hooi et al. (2017). In terms of timeline, the earliest research areas within the field of *H. pylori* vaccines are two research clusters: #2 Heat shock proteins and #4 T cells, which subsequently evolved into #9 Toll-like receptors and #3 *Salmonella*. #5 Proteomics and the subsequent #17 Oral vaccine vehicle and #11 Population attributable fraction evolve together into #1 Neutrophil-activating protein, and then #1 evolves into four clusters: #7 *Lactococcus lactis*, #0 UreB, #10 Population attributable fraction, and #14 Vacuolating cytotoxin vaca, of which #10, #14 and independent #18 Outer membrane vesicle (OMV) evolve together into #0. In addition, the emerging focus areas are #0 UreB, #6 Immunoinformatics, #7 *Lactococcus lactis*, #15 Carrier protein, and #18 Outer membrane vesicle (OMV), which are tightly interrelated, and research about these subjects represents the current research frontier within this field.

Figure 6C shows the top 25 references with the strongest citation burst. The first citation burst in this field occurred in 1995, and between 1995 and 2000 there were one or more bursts each year, each lasting at least 3 years. It is worth noting that “Development of a mouse model of *Helicobacter pylori* infection that mimics human disease” is the paper with the strongest burst (Strength = 26.38), published by Marchetti et al. (1995) in *Science* in 1995, and its burst duration lasts until 2000. “Immunization of BALB/c mice against *Helicobacter Felis* infection with *Helicobacter pylori* urease” by Michetti et al. (1994), published in *Gastroenterology* in 1995, also had a high burst (Strength = 24.55). The next burst of citations after 2000 occurred in 2009. Two citation bursts occurred, respectively, in 2018 and 2019, with two citation bursts in the direction of multi-epitope vaccine research continuing until 2023, indicating that the research related to *H. pylori* vaccine is ongoing.

## 4 Discussion

### 4.1 General information

Our research explores the evolution of *H. pylori* vaccine-related literature from 1993 to 2023 through a comprehensive bibliometric analysis and uses advanced tools such as the VOS viewer and CiteSpace to visually illustrate emerging trends and cutting-edge research focus areas within the field of *H. pylori* vaccine development.

Based on the outcomes of our analysis, there is a general upward trend in the publications and citations in the field related to *H. pylori* vaccines over the past few decades. By analyzing the ratio curve between the citation number of the *H. pylori* vaccine research and the general *H. pylori* research (Figure 1D), it can be found that the development trend of the number of citations in *H. pylori* vaccine-related studies surpasses that in general *H. pylori* studies. Nevertheless, the number of publications per year has not demonstrated a sustained increase. The ratio between the number of publications related to the *H. pylori* vaccine and general *H. pylori* research is unstable (Figure 1C). The trend in terms of the number of publications on *H. pylori* vaccine research has not exceeded that of general *H. pylori* research. Additionally, the most recent clinical trial related to the *H. pylori* vaccine was published by Malfertheiner et al. (2018). We speculate that the development of *H. pylori* vaccines may be less vigorous than the global increase in general biomedical publication activity.

The United States is undoubtedly the most important contributor in this field, both in terms of publications, citation frequency, and national cooperation. Meanwhile, we can also perceive the considerable potential of China and the United Kingdom in this area. Supplementary Table S2 shows that four of the top ten institutions in terms of number of publications are from the United States, while seven of the top ten institutions with the highest citation frequency are also from the United States, demonstrating the substantial financial support from the United States for this area. The considerable investment further reveals that the United States attaches significant importance to the development of *H. pylori* vaccines. *Vaccine* has the highest number of publications, while *Infection And Immunity* is the most cited journal. Thomas F. Meyer, from Germany, is the most published author, while Pierre Michetti from Switzerland is the leading co-cited author, demonstrating their remarkable influence and substantial contribution to the field of *H. pylori* vaccines. The article titled “Immunization of mice with urease vaccine affords protection against *Helicobacter pylori* infection in the absence of antibodies and is mediated by MHC class II-restricted responses” published in *The Journal of Experimental Medicine* by Ermak, T H et al. is the most co-cited and has served as the foundational knowledge for numerous subsequent studies, demonstrating its exceptional contribution to this field.

We also explored the evolutionary tendencies in this field. From a keyword perspective, *H. pylori* and vaccines, as components of the search formulation, formed the basis, center, and key to the research coverage of the whole field. Notably, keywords related to diseases, antigens, and immune responses play an essential role in bridging to other clusters. This indicates that researchers possess distinct research orientations and focuses; however, the core topic and objective remain unchanged. From the perspective of research timelines, earlier studies gave more weight to model construction and immune responses, whereas more recent studies focused more on effective treatment and earlier prevention. In terms of the main topics of the articles, #2 Heat shock proteins and #4 T cells are the larger clusters that appear early. UreB, immunoinformatics, *Lactococcus lactis*, carrier protein, and outer membrane vesicle (OMV) are emerging keyword clusters, and the attention paid to these keyword clusters is still ongoing.

### 4.2 Current status

Several research teams have endeavored to develop an efficient and marketable vaccine targeting H. pylorus, but have not been successful. Currently, most vaccines in development are in the early stages. Despite the numerous vaccine candidates undergoing clinical trials, only a few have demonstrated promising immunization outcomes.

In 1996, Kreiss et al. conducted a pioneering clinical trial and found that despite oral administration of a recombinant *H. pylori* urease vaccine, all volunteers remained infected with the bacterium, highlighting the need for further research (Kreiss et al., 1996). Subsequently, Michetti et al. discovered that the combination of *H. pylori* urease and *Escherichia coli* heat-labile enterotoxin (LT) could considerably reduce the bacterial load in the stomach, but fail to eliminate *H. pylori* infection (Michetti et al., 1999). The addition of an effective adjuvant can significantly improve immunization. Similar conclusions were obtained by Kotloff et al. using a formalin-inactivated, oral *H. pylori* whole-cell (HWC) vaccine administered with LT (R192G) (Kotloff et al., 2001). Malfertheiner et al. developed an innovative *H. pylori* vaccine containing three key virulence-associated antigens (VacA, CagA, and NAP) and an aluminium hydroxide adjuvant. While this intramuscular vaccine demonstrated satisfactory safety and immunogenicity, a rigorous phase 1/2 clinical trial revealed that it failed to offer additional protection against *H. pylori* infection (Malfertheiner et al., 2018; Malfertheiner et al., 2008). Furthermore, researchers explored the potential of utilizing *Salmonella typhi* as a vector for *H. pylori* vaccine delivery. However, some clinical trials have demonstrated that the utilization of *Salmonella typhi* as a carrier for *H. pylori* vaccine delivery has failed to exhibit satisfactory protective effects, but these trials have provided valuable insights into T cell-mediated immunity against *H. pylori* infection, paving the way for future vaccine advancements (Bumann et al., 2001; Metzger et al., 2004; Aebischer et al., 2008).

*H. pylori* colonization typically takes place in early childhood, and vaccines customized for children are crucial for the early prevention of *H. pylori* (Duan et al., 2023; Zhou et al., 2023). A promising recombinant oral vaccine against *H. pylori*, which incorporates both UreB and LTB components, has completed phase III clinical trials, demonstrating the potential to significantly decrease the *H. pylori* infection rate among children (Zeng et al., 2015). One year after vaccination, the incidence decreased by 71.8% (95% CI 48.2% ~ 85.6%), and the protection rate decreased to 55% after 2 years.

In animal models, the most recent research focuses on a M cell-targeting recombinant *L. lactis* vaccine. Based on the promising outcomes in mice (Guo et al., 2022; Zhang et al., 2024), further research and clinical trials are needed to assess the effectiveness of the M cell-targeting recombinant *L. lactis* vaccine in controlling *H. pylori* infection in humans. Additionally, the use of nanoparticles as a platform for developing vaccines against *H. pylori* also shows potential (Safarov et al., 2019; Skakic et al., 2023). Successfully synthesized *H. pylori* urease A subunit nanocapsules and found that vaccinating mice with larger nanocapsules and adjuvant combinations can significantly reduce colonization. Multivalent subunit vaccines containing CagA, VacA, and NAP have been proven to be effective in reducing *H. pylori* infection (Huang et al., 2024). Continued research into multivalent subunit vaccines containing CagA, VacA, NAP, and other components is also important for developing effective strategies to combat *H. pylori* infection.

Collectively, these studies highlight the potential for novel vaccine candidates and platforms to address *H. pylori* infection. The potential for this area of research is significant and further research and development is essential to improve our understanding of how to effectively prevent and eradicate this common bacterial infection.

### 4.3 Major challenges

In spite of all efforts, producing an efficient and marketable vaccine against *H. pylori* infection remains a complex challenge. This bacterium has developed a number of mechanisms to adapt to the hostile gastric environment, allowing it to evade the immune system mechanisms and establish chronic infections (Malfertheiner et al., 2022; Toh and Wilson, 2020). A large number of virulence factors, such as CagA, VacA, and outer membrane proteins like BabA, and SabA, are encoded by *H. pylori* to participate in these processes (Toh and Wilson, 2020). Furthermore, *H. pylori*’s genome exhibits a high degree of variation, with 20–30% genomic differences between strains, due to its rapid mutation and recombination rates (Maleki Kakelar et al., 2019). Therefore, a useful vaccine should be immunogenic sufficient that target the diverse strains of *H. pylori*. Numerous studies have indicated that vaccines relying solely on a single antigen are inadequate in combating *H. pylori*, while multivalent subunit vaccines have proven to be more effective in providing protection (Zeng et al., 2015; Zhang et al., 2024; Huang et al., 2024). Several studies have comprehensively summarized the bacterial antigens with vaccine production potential identified during the long journey of *H. pylori* vaccine research (Dos Santos Viana et al., 2021; Li et al., 2023).

Another challenge is to identify adjuvants that are both human-safe and capable of eliciting a targeted immune response while minimizing the required dosage of immune agent (Longet et al., 2019). A review has provided a comprehensive summary of existing adjuvants (Li et al., 2023). Recent studies have investigated novel adjuvants as potential replacements for current ones to solve the existing limitations, and outer membrane vesicles (OMVs) may be a promising candidate adjuvant (Song et al., 2020).

Despite the high prevalence and substantial public health burden of *H. pylori*, the development of the *H. pylori* vaccine has been relatively slow. The steps required to develop a new vaccine are time-consuming and costly, and factors such as the urgency of the demand for a new vaccine and public interest can influence the speed at which a vaccine enters the market. In this context, *H. pylori* vaccines differ from other high-priority infectious disease vaccines, such as COVID-19. Given the escalating issue of antibiotic resistance, developing a vaccine targeting *H. pylori* may be the most viable long-term solution for preventing this infection in the future.

### 4.4 Limitations

Our study is the first bibliometric visualization of *H. pylori* vaccine-related research over the past decades. However, there are inevitable limitations in this study. Firstly, the literature search is limited to the WoSCC database. In spite of the high quality and credibility of the WoSCC database, the search approach using a single database may still miss some articles. And this study only included English language articles and reviews, which may have excluded some papers in other languages, as well as early access and proceedings papers. Some of these exclusions might give rise to biased outcomes. Nevertheless, given the extensive time span of our study and the considerable number of included studies, we still maintain that our study holds considerable credibility. Secondly, the citation frequency of recently published papers cannot be directly compared to that of previously published ones. Supplementary Table S6 lists the top 10 most frequently cited references on *H. pylori* vaccines. However, the publication time gap of the articles is significant, and a direct comparison of citation frequency will generate biased results. Hence, we plotted the annual citation times of the top 10 highly cited literatures since their publication, as shown in Supplementary Figure S2. Through Supplementary Figure S2, it is possible for us to clearly observe the annual citation trend of major articles. However, we are unable to present the annual citation circumstances of all literatures, which constitutes the deficiency of our study. Thirdly, the data presented in this study might be inconsistent in various aspects; for instance, the same institution could have employed diverse names at distinct periods.

## 5 Conclusion

This study is the first bibliometric analysis focusing on *H. pylori* vaccines. Using the WoSCC database, we downloaded 1,199 original publications related to *H. pylori* vaccines from 1993 to 2023. To gain insight into the data, we use CiteSpace and VOS viewer software to generate detailed cluster analysis graphs. These graphs provide a holistic and objective perspective on the current status, trends, and frontiers in *H. pylori* vaccine research. Over the past decades, the United States, China, and the United Kingdom being the core research countries, have made substantial contributions to this field. *Vaccine* and *Infection And Immunity* are two of the most influential journals in this field. Thomas F. Meyer and Pierre Michetti are important scholars in this field. Our analysis also highlights that developing effective treatment strategies and multi-epitope vaccines continue to be prominent research areas in *H. pylori* vaccines field. In summary, our study provides valuable insights into the current state of *H. pylori* vaccine research and identifies promising directions for future exploration. We anticipate that this study will provide valuable data for relevant researchers and contribute to the subsequent development of *H. pylori* vaccines.

## Data Availability

Publicly available datasets were analyzed in this study. This data can be found here: Web of Science Core Collection.
