# Supplementary material for: Bibliometric analysis of Helicobacter pylori vaccine development from 1993 to 2023
Source: Front Microbiol. 2025 Mar 17;16:1479195. doi: 10.3389/fmicb.2025.1479195 (PMC11955499; doi:10.3389/fmicb.2025.1479195)
Supplement: Supplementary file 1 [file Data_Sheet_1.docx]

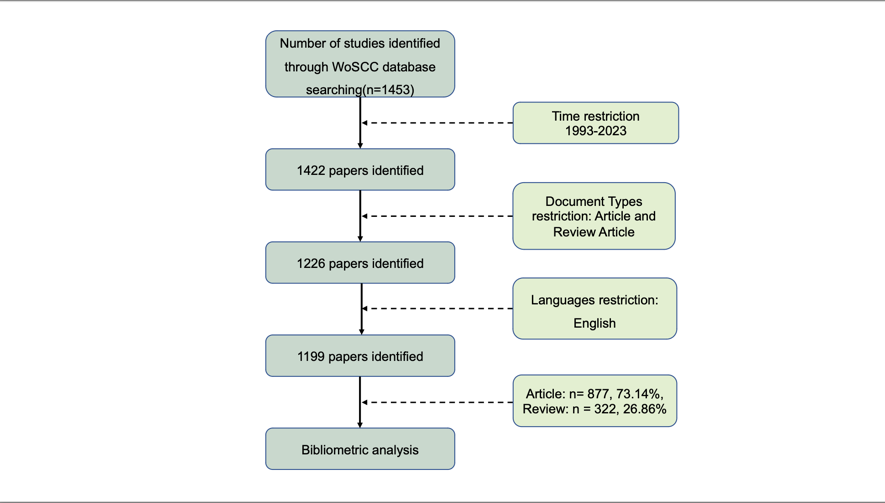
Figure S1 Flow chart of literature screening.

We searched the Web of Science Core Collection (WOSCC) database on June 3, 2024, using the following search formula: (TS = (“*Helicobacter pylori*” OR “*Campylobacter pylori*” OR “*H. pylori*” OR “*Campylobacter pylori subsp. Pylori*” OR “*Campylobacter pyloridis*” OR “*Campylobacter pylori*”)) AND TS = (Vaccine). A total of 1199 original English-language articles on *H. pylori* and vaccines are retrieved from 1993 to 2023, encompassing both articles and reviews.


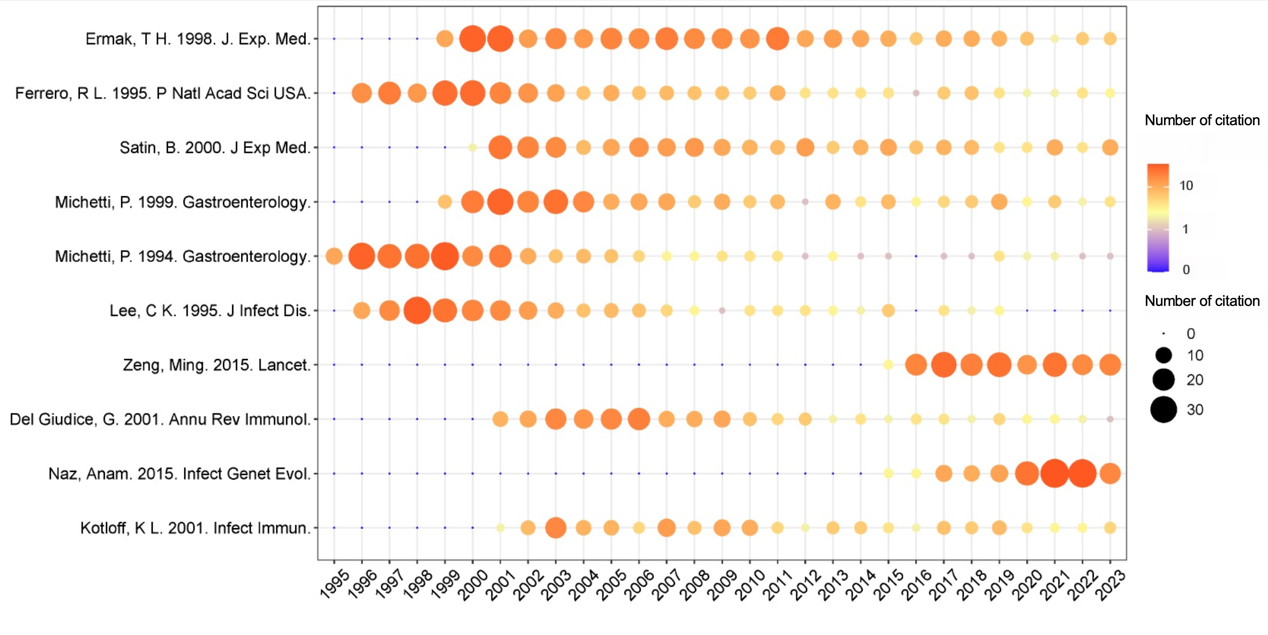


Figure S2 Annual citations of top10 highly cited references.

The annual number of citations for papers with a high citation score, where the size and color of the circles denote the citation of the literature. The larger circles and colors ranging from blue to red indicate higher citation rates and greater influence within the field.

Table S1 Top 10 countries in terms of number of publications, frequency of citations, and total link strength.

| **Rank** | **Country** | **Documents** | **Rank** | **Country** | **Citations** | **Rank** | **Country** | **Total Link Strength** |
| --- | --- | --- | --- | --- | --- | --- | --- | --- |
| **1** | United States | 327 | **1** | United States | 18360 | **1** | United States | 194 |
| **2** | China | 236 | **2** | Italy | 6071 | **2** | United Kingdom | 90 |
| **3** | United Kingdom | 92 | **3** | United Kingdom | 4720 | **3** | China | 67 |
| **4** | Germany | 86 | **4** | China | 4561 | **4** | Australia | 61 |
| **5** | Australia | 76 | **5** | Germany | 4437 | **5** | Italy | 59 |
| **6** | Italy | 76 | **6** | Sweden | 3329 | **6** | Germany | 56 |
| **7** | Iran | 71 | **7** | Switzerland | 2249 | **7** | France | 43 |
| **8** | Sweden | 58 | **8** | France | 2220 | **8** | Pakistan | 42 |
| **9** | India | 49 | **9** | Australia | 2205 | **9** | Sweden | 41 |
| **10** | Canada &Switzerland | 42 | **10** | South Korea | 1394 | **10** | Canada | 41 |

Table S2 Top 10 institutions in terms of number of articles issued and frequency of citations.

| **Rank** | **Institution** | **Documents** | **Original**  **Country** | **Rank** | **Institution** | **Citations** | **Original**  **Country** |
| --- | --- | --- | --- | --- | --- | --- | --- |
| **1** | Univ Maryland | 36 | United States | **1** | Stanford Univ | 1968 | United States |
| **2** | Univ Gothenburg | 34 | Sweden | **2** | Univ Maryland | 1716 | United States |
| **3** | Univ Melbourne | 26 | Australia | **3** | Max Planck Inst Infect Biol | 1529 | Germany |
| **4** | Max Planck Inst Infect Biol | 22 | Germany | **4** | Univ Gothenburg | 1159 | Sweden |
| **5** | Harvard Univ | 22 | United States | **5** | Oravax Inc | 1146 | United States |
| **6** | China Pharmaceut Univ | 22 | China | **6** | Washington Univ | 1081 | United States |
| **7** | Third Mil Med Univ | 22 | China | **7** | Vanderbilt Univ | 1000 | United States |
| **8** | Case Western Reserve Univ | 17 | United States | **8** | Umea Univ | 981 | Sweden |
| **9** | Baylor Coll Med | 15 | United States | **9** | Baylor Coll Med | 941 | United States |
| **10** | Univ Lausanne | 15 | Switzerland | **10** | Case Western Reserve Univ | 929 | United States |

Table S3 Top 10 author and co-cited authors related to the *H. pylori* vaccine.

| **Rank** | **Author** | **Documents** | **Country** | **Author** | **Co-citations** | **Country** | **Author** | **Total Link Strength** | **Country** |
| --- | --- | --- | --- | --- | --- | --- | --- | --- | --- |
|  |  |  |  |  |  |  |  |  |  |
| 1 | Thomas F. Meyer | 28 | Germany | Pierre Michetti | 309 | Switzerland | Pierre Michetti | 7855 | Switzerland |
| 2 | R.Rappuoli | 23 | United Kingdom | A Lee | 271 | Australia | Richard L. Ferrero | 6875 | Switzerland |
| 3 | Philip Sutton | 22 | Australia | Peter Malfertheiner | 269 | Germany | Thomas G. Blanchard | 6834 | United States |
| 4 | Quan-Ming Zou | 22 | China | Philip Sutton | 269 | Australia | Kathryn A. Eaton | 6803 | United States |
| 5 | Pierre Michetti | 20 | United States | David Y. Graham | 261 | United States | Steven J. Czinn | 6446 | United States |
| 6 | Steven J. Czinn | 20 | Switzerland | Richard L. Ferrero | 260 | Australia | David Graham | 6408 | United States |
| 7 | Tao Xi | 18 | China | M. J. Blaser | 224 | United States | A Lee | 6356 | Australia |
| 8 | Yingying Xing | 18 | China | Thomas Ermak | 224 | United States | Philip Sutton | 6274 | Australia |
| 9 | Anton Aebischer | 17 | Germany | Steven J. Czinn | 223 | United States | Thomas Ermak | 5982 | United States |
| 10 | Dirk Bumann | 17 | Germany | Kathryn A. Eaton | 223 | United States | CK Lee | 5440 | United States |

Table S4 The number of publications, IF (JCR2023), and JCR quartile of the top 10 journals and co-cited-journals.

| **Rank** | **Journal** | **Publications** | **IF**  **(JCR2023)** | **JCR**  **quartile** | **Co-cited-journal** | **Citations** | **IF**  **(JCR2023)** | **JCR**  **quartile** |
| --- | --- | --- | --- | --- | --- | --- | --- | --- |
|  |  |  |  |  |  |  |  |  |
| 1 | Vaccine | 82 | 4.5 | Q2 | Infection And Immunity | 6001 | 2.9 | Q2 |
| 2 | Infection And Immunity | 74 | 2.9 | Q2 | Vaccine | 3698 | 4.5 | Q2 |
| 3 | Helicobacter | 67 | 4.3 | Q2 | Gastroenterology | 2689 | 25.7 | Q1 |
| 4 | World Journal of Gastroenterology | 39 | 4.3 | Q2 | P Natl Acad Sci USA | 1815 | 9.4 | Q1 |
| 5 | Plos One | 23 | 2.9 | Q1 | Journal of Immunology | 1785 | 3.6 | Q2 |
| 6 | Frontiers in Immunology | 21 | 5.7 | Q1 | Helicobacter | 1416 | 4.3 | Q2 |
| 7 | Applied Microbiology And Biotechnology | 16 | 3.9 | Q2 | Lancet | 1321 | 98.4 | Q1 |
| 8 | Scientific Reports | 16 | 3.8 | Q1 | Gut | 1320 | 23.0 | Q1 |
| 9 | Current Opinion in Gastroenterology | 15 | 2.6 | Q2 | J Infect Dis | 1289 | 5.0 | Q1 |
| 10 | Gastroenterology | 15 | 25.7 | Q1 | Science | 1159 | 44.7 | Q1 |

Table S5 Top 20 keywords with the highest occurrence times and their total link strength.

| **Rank** | **Keyword** | **Occurrences** | **Total link strength** | **Rank** | **Keyword** | **Occurrences** | **Total link strength** |
| --- | --- | --- | --- | --- | --- | --- | --- |
|  |  |  |  |  |  |  |  |
| 1 | H. pylori | 743 | 5067 | 11 | expression | 114 | 904 |
| 2 | infections | 433 | 3053 | 12 | protection | 106 | 939 |
| 3 | vaccines | 318 | 2489 | 13 | antigen | 102 | 808 |
| 4 | mouse model | 285 | 2294 | 14 | immunogenicity | 99 | 835 |
| 5 | immune responses | 222 | 1665 | 15 | protein | 93 | 676 |
| 6 | urease | 167 | 1359 | 16 | identification | 84 | 645 |
| 7 | immunization | 162 | 1325 | 17 | antibody | 81 | 640 |
| 8 | oral immunization | 162 | 1354 | 18 | adjuvants | 79 | 706 |
| 9 | escherichia-coli | 128 | 854 | 19 | virulence factor | 73 | 537 |
| 10 | gastric cancer | 117 | 900 | 20 | genome sequence | 68 | 487 |

Table S6 Top 10 high-cited references related to H. pylori vaccine.

| **Rank** | **Authors** | **Article Title** | **Source Title** | **Citations** | **Year** | **Document Type** | **DOI** |
| --- | --- | --- | --- | --- | --- | --- | --- |
| 1 | Ermak, T H et al. | Immunization of mice with urease vaccine affords protection against Helicobacter pylori infection in the absence of antibodies and is mediated by MHC class II-restricted responses | *J. Exp. Med.* | 344 | 1998 | Article | 10.1084/jem.188.12.2277 |
| 2 | Ferrero, R L et al. | The GroES homolog of Helicobacter pylori confers protective immunity against mucosal infection in mice | *P Natl Acad Sci USA* | 256 | 1995 | Article | 10.1073/  pnas.92.14.6499 |
| 3 | Satin, B et al. | The neutrophil-activating protein (HP-NAP) of Helicobacter pylori is a protective antigen and a major virulence factor | *The Journal of experimental medicine* | 245 | 2000 | Article | 10.1084/  jem.188.12.2277 |
| 4 | Michetti, P et al. | Oral immunization with urease and Escherichia coli heat-labile enterotoxin is safe and immunogenic in Helicobacter pylori-infected adults | *Gastroenterology* | 242 | 1999 | Article | 10.1016/S0016-5085  (99)70063- |
| 5 | Michetti, P et al. | Immunization of BALB/c mice against Helicobacter felis infection with Helicobacter pylori urease | *Gastroenterology* | 234 | 1994 | Article | 10.1016/0016-5085  (94)90224-0 |
| 6 | Cynthia K.Lee  et al. | Oral immunization with recombinant Helicobacter pylori urease induces secretory IgA antibodies and protects mice from challenge with Helicobacter felis | *The Journal of Infectious Diseases* | 206 | 1995 | Article | 10.1093/  infdis/172.1.161 |
| 7 | Zeng, Ming et al. | Efficacy, safety, and immunogenicity of an oral recombinant Helicobacter pylori vaccine in children in China: a randomised, double-blind, placebo-controlled, phase 3 trial | *Lancet* | 169 | 2015 | Article | 10.1016/S0140-6736(15)  60310-5 |
| 8 | Del Giudice, G et al. | The design of vaccines against Helicobacter pylori and their development | *Annual review of immunology* | 172 | 2001 | Review | 10.1146/  annurev.immunol.19.1.523 |
| 9 | Naz, Anam et al. | Identification of putative vaccine candidates against Helicobacter pylori exploiting exoproteome and secretome: A reverse vaccinology based approach | *Infect Genet Evol* | 150 | 2015 | Article | 10.1016/  j.meegid.2015.03.027 |
| 10 | Kotloff, K L et al. | Safety and immunogenicity of oral inactivated whole-cell Helicobacter pylori vaccine with adjuvant among volunteers with or without subclinical infection | *The Infection and immunity* | 153 | 2001 | Article | 10.1128/  IAI.69.6.3581-3590.2001 |
